# A new species of the *Rana japonica* group (Anura, Ranidae, *Rana*) from China, with a taxonomic proposal for the *R. johnsi* group

**DOI:** 10.3897/zookeys.942.46928

**Published:** 2020-06-18

**Authors:** Han Wan, Zhi-Tong Lyu*, Shuo Qi, Jian Zhao, Pi-Peng Li, Ying-Yong Wang

**Affiliations:** 1 State Key Laboratory of Biocontrol/ The Museum of Biology, School of Life Sciences, Sun Yat-sen University, Guangzhou 510275, China; 2 School of Ecology, Sun Yat-sen University, Guangzhou 510006, China; 3 Institute of herpetology, Shenyang Normal University, Shenyang 110034, China; 4 Shenzhen Shuanghuamu Biological Technology Co., Ltd., Shenzhen 51800, China

**Keywords:** morphology, phylogeny, *Rana jiulingensis* sp. nov., *Rana sangzhiensis*, *Rana zhengi*

## Abstract

*Rana jiulingensis***sp. nov.**, a new species from Hunan and Jiangxi, southeastern China, is described. The new species is assigned to the *R. japonica* group. The clade comprising *R. jiulingensis***sp. nov.** and *R. dabieshanensis* from Anhui is the sister taxon of *R. omeimontis* from Sichuan. *Rana jiulingensis***sp. nov.** can be distinguished by the significant divergences in the 16S and COI genes, and the combination of following morphological characters: body size medium, SVL 48.3–57.8 mm in adult males and 48.2–57.5 mm in adult females; dorsolateral fold straight; digits without circummarginal grooves; dorsal skin smooth; tibio-tarsal articulation reaching forward beyond the tip of snout; heels overlapping; webbing formula of toes: I 1⅓ – 2 II 1⅓ – 2⅓ III 1½ – 2⅔ IV 3 – 1⅓ V; absence of vocal sacs in males; and presence of creamy white nuptial pad with tiny hoar spines on the finger I and reddish tubercles on loreal and temporal regions in breeding males. Furthermore, based on our results and the previous literature, *R. zhengi* is synonymized with *R. sangzhiensis*, and a new species group, the *Rana johnsi* group, is proposed for the *R. johnsi* and *R. sangzhiensis*. Currently, the Rana contains 41 recognized species, and the phylogenetic placements of several species remain unresolved.

## Introduction

As the type genus of the family Ranidae Batsch, 1796, the concept of the true-frog genus *Rana* Linnaeus, 1758 has been discussed for a long time (Frost 2020). In a recent phylogenetic analysis (Yuan et al. 2016), *Rana* sensu lato was considered to be composed of nine clades, namely the subgenera *Rana*, *Amerana* Dubois, 1992, *Liuhurana* Fei, Ye, Jiang, Dubois & Ohler, 2010, *Aquarana* Dubois, 1992, *Lithobates* Fitzinger, 1843, *Zweifelia* Dubois, 1992, *Pantherana* Dubois, 1992, *Pseudorana* Fei, Ye & Huang, 1990, and an unnamed monotypic clade containing *R. sylvatica* (LeConte, 1825). However, this classification is still controversial, especially for the recognitions of the genera *Lithobates* and *Pseudorana* (Frost 2020). Nevertheless, the subgenusRana, which is currently well recognized, contains 41 known species distributed from Europe to southeastern Asia. Among them, 23 species occur in China (AmphibiaWeb 2019). Recent researches on this subgenus have discovered new species from China and revised several taxonomic errors, indicating that the diversity and taxonomy of the subgenusRana are still insufficiently understood (Yan et al. 2011; Zhou et al. 2015, 2017; Yuan et al. 2016; Wang et al. 2017; Yang et al. 2017; Zhao et al. 2017).

Based on morphological comparisons and geographical conditions, Fei et al. (2009) proposed three species groups for the Chinese species of the subgenusRana: *R. longicrus* group, *R. chensinensis* group, and *R. amurensis* group. Subsequent phylogenetic analyses have revised several memberships of these groups (Yan et al. 2011; Zhou et al. 2015, 2017; Yuan et al. 2016; Wang et al. 2017; Zhao et al. 2017), and the nomenclature of the *R. longicrus* group was replaced by the *R. japonica* group (Yang et al. 2017). Currently, 16 Chinese species are recognized as members of the three species groups. The *R. japonica* group contains nine species: R. (R.) chaochiaoensis Liu, 1946; R. (R.) chevronta Hu & Ye, 1978; R. (R.) culaiensis Li, Lu & Li, 2008; R. (R.) dabieshanensis Wang, Qian, Zhang, Guo, Pan, Wu, Wang & Zhang, 2017; R. (R.) hanluica Shen, Jiang & Yang, 2007; R. (R.) jiemuxiensis Yan, Jiang, Chen, Fang, Jin, Li, Wang, Murphy, Che & Zhang, 2011; R. (R.) longicrus Stejneger, 1898; R. (R.) omeimontis Ye & Fei, 1993; and R. (R.) zhenhaiensis Ye, Fei & Matsui, 1995. The *R. chensinensis* group contains four species: R. (R.) chensinensis David, 1875; R. (R.) dybowskii Günther, 1876; R. (R.) huanrenensis Liu, Zhang & Liu, 1993; and R. (R.) kukunoris Nikolskii, 1918. The *R. amurensis* group has three species: R. (R.) amurensis Boulenger, 1886; R. (R.) coreana Okada, 1928; and R. (R.) luanchuanensis Zhao & Yuan, 2017. However, species groups have not yet been proposed to accommodate the remaining seven species: R. (R.) arvalis Nilsson, 1842; R. (R.) asiatica Bedriaga, 1898; R. (R.) maoershanensis Lu, Li & Jiang, 2007; R. (R.) sauteri Boulenger, 1909; R. (R.) johnsi Smith, 1921; R. (R.) sangzhiensis Shen, 1986; and R. (R.) zhengi Zhao, 1999.

**Figure 1.**
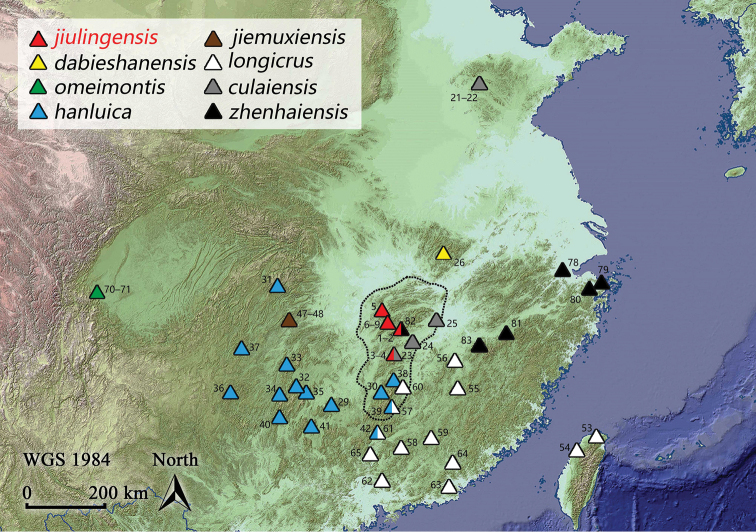
Collecting localities of *Rana* samples used in this study. Dotted line shows the Luoxiao Range, where five *Rana* species are recorded. Numbers correspond to the ID in Table 1.

During herpetofaunal surveys in the Luoxiao Range, which is situated between the Jiangxi and Hunan provinces (Fig. 1), a series of *Rana* specimens was collected which can be assigned to the *R. japonica* group based on morphological characteristics. However, detailed examination of these specimens showed significant differences from all known congeners. Additional molecular analysis well supported the morphological identifications, demonstrating that these specimens formed an unnamed lineage within the *R. japonica* group. Therefore, we describe this series of specimens as a new species. Additionally, as revealed from our results and the previous literature, we suggest that *R. zhengi* should be synonymized with *R. sangzhiensis*, and we also propose a new species group, the *Rana johnsi* group, for the species *R. johnsi* and *R. sangzhiensis*.

## Materials and methods

### Sampling and morphological analyses

Eight unnamed specimens were collected from multiple localities of Jiangxi and Hunan provinces. All specimens were fixed in 10% buffered formalin, later transferred to 70% ethanol, and deposited in the Museum of Biology, Sun Yat-sen University (**SYS**) and Chengdu Institute of Biology, Chinese Academy of Sciences (**CIB**), PR China. External measurements were made for the unnamed specimens with digital calipers (Neiko 01407A Stainless Steel 6-Inch Digital Caliper, USA) to the nearest 0.1 mm. These measurements are as follows:

**SVL** snout–vent length (from tip of snout to posterior margin of vent);

**HL** head length (from tip of snout to the articulation of the jaw);

**HW** head width (head width at the commissure of the jaws);

**SL** snout length (from tip of snout to the anterior corner of the eye);

**IN** internasal distance (distance between nares);

**IO** interorbital distance (minimum distance between upper eyelids);

**ED** eye diameter (from the anterior corner of the eye to posterior corner of the eye);

**TD** tympanum diameter (horizontal diameter of tympanum);

**TED** tympanum–eye distance (from anterior edge of tympanum to posterior corner of the eye);

**HND** hand length (from the proximal border of the outer palmar tubercle to the tip of digit III);

**RAD** radio-ulna length (from the flexed elbow to the proximal border of the outer palmar tubercle);

**FTL** foot length (from distal end of shank to the tip of digit IV);

**TIB** tibial length (from the outer surface of the flexed knee to the heel).

The morphological description follows the consistent definition by Fei et al. (2009). Sex and age were determined by examining the gonads. Webbing formula was based on Savage (1975). Comparison characters of known congeners were obtained from the literature (Stejneger 1898; Liu 1946; Liu et al. 1993; Ye et al. 1993, 1995; Lu et al. 2007; Shen et al. 2007; Li et al. 2008; Fei et al. 2009, 2012; Yan et al. 2011; Wang et al. 2017; Zhao et al. 2017) and 80 examined museum specimens listed in the Appendix 1.

### DNA Extraction, PCR amplification, and sequencing

A total of 56 muscular samples of *Rana* were used, encompassing nine samples of the undescribed specimens, and 47 samples from 12 recognized species. All samples were attained from euthanasia specimens and then preserved in 95% ethanol and stored at –40 °C. Genomic DNA were extracted from muscle tissue samples, using DNA extraction kit from Tiangen Biotech (Beijing) Co., Ltd. Two mitochondrion genes, namely partial 16S ribosomal RNA gene (16S) and partial cytochrome c oxidase 1 gene (COI), were amplified. Primers used for 16S were L3975 (5'-CGCCTGTTTACCAAAAACAT-3') and H4551 (5'-CCGGTCTGAACTCAGATCACGT-3') following Simon et al. (1994), and L2A (5'-CCAAACGAGCCTAGTGATAGCTGGTT-3') and H10 (5'-TGATTACGCTACCTTTGCACGGT-3') following Chen et al. (2013), for COI were dgLCO (5'-GGTCAACAAATCATAAAGAYATYGG-3') and dgHCO (5'-AAACTTCAGGGTGACCAAARAAYCA-3') following Meyer et al. (2005). PCR amplifications were processed with the cycling conditions that initial denaturing step at 95 °C for 4 min, 35 cycles of denaturing at 94 °C for 40 s, annealing at 53 °C (for 16S) / 48 °C (for COI) for 40 s and extending at 72 °C for 60 s, and a final extending step at 72 °C for 10 min. PCR products were purified with spin columns and then sequenced with both forward and reverse primers using BigDye Terminator Cycle Sequencing Kit per the guidelines, on an ABI Prism 3730 automated DNA sequencer by Shanghai Majorbio Bio-pharm Technology Co., Ltd. All sequences were deposited in GenBank (Table 1).

**Table 1.** Localities, voucher information, and GenBank numbers for all samples of the genus *Rana* used in this study (* = type localities).

ID	Species	Localities	Voucher no.	16S	COI
**1**	*R. jiulingensis*	China: Jiangxi: Mt Guanshan *	SYS a005519	MT408985	MT418647
**2**	*R. jiulingensis*	China: Jiangxi: Mt Guanshan *	SYS a006999	MT408994	MT418656
**3**	*R. jiulingensis*	China: Jiangxi: Mt Wugong	SYS a002584	MT408964	MT418626
**4**	*R. jiulingensis*	China: Jiangxi: Mt Wugong	SYS a002585	MT408965	MT418627
**5**	*R. jiulingensis*	China: Hunan: Mt Mufu	SYS a005511	MT408984	MT418646
**6**	*R. jiulingensis*	China: Hunan: Mt Dawei	SYS a006451	MT408989	MT418651
**7**	*R. jiulingensis*	China: Hunan: Mt Dawei	SYS a006494	MT408990	MT418652
**8**	*R. jiulingensis*	China: Hunan: Mt Dawei	SYS a006495	MT408991	MT418653
**9**	*R. jiulingensis*	China: Hunan: Mt Dawei	SYS a006496	MT408992	MT418654
**10**	*R. amurensis*	China: Heilongjiang: Taiyang Island	SYNU 11100267	KF020589	KF020603
**11**	*R. amurensis*	China: Liaoning, Zhangwu	SYNU 11100268	KU343216	KU343216
**12**	*R. arvalis*	Germany: Lower Saxony	No voucher	AY147938	/
**13**	*R. asiatica*	China: Xinjiang: 47tuan	KIZ XJ0251	KX269200	/
**14**	*R. chaochiaoensis*	China: Sichuan: Zhaojue *	SYS a001815	MT409007	MT418669
**15**	*R. chaochiaoensis*	China: Sichuan: Zhaojue *	SYS a001816	MT408957	MT418619
**16**	*R. chensinensis*	China: Shaanxi: Huxian *	KIZ RD05SHX01	KX269186	JF939080
**17**	*R. chensinensis*	China: Henan: Mt Yawu	SYS a002392	MT408962	MT418624
**18**	*R. chensinensis*	China: Henan: Mt Yawu	SYS a002393	MT408963	MT418625
**19**	*R. coreana*	South Korea	MMS 223	KX269202	MF149928
**20**	*R. coreana*	China: Shandong: Mt Kunyu	SYNU 08090641	MT409004	MT418666
**21**	*R. culaiensis*	China: Shandong: Mt Culai *	KIZ SD080501	KX269190	JF939082
**22**	*R. culaiensis*	China: Shandong: Mt Culai *	SYNU 08090549	MT409006	MT418668
**23**	*R. culaiensis*	China: Jiangxi: Mt Wugong	SYS a002634	MT408966	MT418628
**24**	*R. culaiensis*	China: Jiangxi: Shanggao	SYS a002641	MT408967	MT418629
**25**	*R. culaiensis*	China: Jiangxi: Mt Meiling	SYS a004239	MT408971	MT418633
**26**	*R. dabieshanensis*	China: Anhui: Dabie Mountains area *	AHU 2016R001	MF172963	/
**27**	*R. dybowskii*	Russia: Primorye: Khasanskii	MSUZP-IVM-1d	KX269188	/
**28**	*R. dybowskii*	China: Jilin: Mt Laoling	SYNU 11070163	MT409005	MT418667
**29**	*R. hanluica*	China: Hunan: Mt Yangming *	SYS a001137	MT408956	MT418618
**30**	*R. hanluica*	China: Hunan: Mt Bamian	SYS a004086	MT408969	MT418631
**31**	*R. hanluica*	China: Hunan: Mt Badagong	SYS a004298	MT408973	MT418635
**32**	*R. hanluica*	China: Hunan: Mt Yunshan	SYS a004359	MT408977	MT418639
**33**	*R. hanluica*	China: Hunan: Mt Xuefeng	SYS a007216	MT408999	MT418661
**34**	*R. hanluica*	China: Hunan: Suining	SYS a007250	MT409000	MT418662
**35**	*R. hanluica*	China: Hunan: Mt Shunhuang	SYS a007259	MT409001	MT418663
**36**	*R. hanluica*	China: Guizhou: Mt Leigong	SYS a002233	MT408959	MT418621
**37**	*R. hanluica*	China: Guizhou: Mt Fanjing	SYS a004346	MT408976	MT418638
**38**	*R. hanluica*	China: Jiangxi: Mt Jinggang	SYS a004033	MT408968	MT418630
**39**	*R. hanluica*	China: Jiangxi: Mt Qiyun	SYS a004087	MT408970	MT418632
**40**	*R. hanluica*	China: Guangxi: Longsheng	SYS a002286	MT408960	MT418622
**41**	*R. hanluica*	China: Guangxi: Mt Dupangling	SYS a005087	MT408980	MT418642
**42**	*R. hanluica*	China: Guangdong: Renhua	SYS a007100	MT408998	MT418660
**43**	*R. huanrenensis*	China: Liaoning: Huanren *	SYNU 07040035	KF204642	KX139725
**44**	*R. huanrenensis*	China: Liaoning: Huanren *	y-d20130058	KT588071	KT588071
**45**	*R. japonica*	Japan: Isumi-shi: Chiba Prefecture	KIZ YPX11775	KX269220	JF939101
**46**	*R. japonica*	Japan: Isumi-shi: Chiba Prefecture	NNRj	AB728192	/
**47**	*R. jiemuxiensis*	China: Hunan: Jiemuxi *	SYS a004318	MT408975	MT418637
**48**	*R. jiemuxiensis*	China: Hunan: Jiemuxi *	SYS a004319	MT409008	MT418670
**49**	*R. johnsi*	Vietnam: Lam Dong: Loc Bao	ABV 00203	KX269182	/
**50**	*R. kukunoris*	China: Qinghai: Qinghai Lake *	KIZ CJ06102001	KX269185	JF939073
**51**	*R. kukunoris*	China: Sichuan: Hongyuan	SYS a006652	MT409009	MT418671
**52**	*R. kukunoris*	China: Sichuan: Hongyuan	SYS a006653	MT408993	MT418655
**53**	*R. longicrus*	China: Taiwan:Taipei *	Not given	AB058881	/
**54**	*R. longicrus*	China: Taiwan: Miaoli: Xiangtianhu	NMNS 15022	KX269189	/
**55**	*R. longicrus*	China: Fujian: Mt Yashu	SYS a005905	MT408987	MT418649
**56**	*R. longicrus*	China: Jiangxi: Mt Magu	SYS a007038	MT408996	MT418658
**57**	*R. longicrus*	China: Jiangxi: Mt Qiyun	SYS a002355	MT408961	MT418623
**58**	*R. longicrus*	China: Jiangxi: Mt Jiulian	SYS a004487	MT408978	MT418640
**59**	*R. longicrus*	China: Jiangxi: Mt Sanbai	SYS a005892	MT408986	MT418648
**60**	*R. longicrus*	China: Jiangxi: Suichuan	SYS a007097	MT408997	MT418659
**61**	*R. longicrus*	China: Guangdong: Renhua	SYS a000735	MT408954	MT418616
**62**	*R. longicrus*	China: Guangdong: Mt Nankun	SYS a000754	MT408955	MT418617
**63**	*R. longicrus*	China: Guangdong: Pu’ning	SYS a004605	MT408979	MT418641
**64**	*R. longicrus*	China: Guangdong: Mt Tonggu	SYS a005218	MT408981	MT418643
**65**	*R. longicrus*	China: Guangdong: Yingde	SYS a007519	MT409003	MT418665
**66**	*R. maoershanensis*	China: Guangxi: Mt Maoershan *	SYNU 08030061	HQ228162	/
**67**	*R. maoershanensis*	China: Guangxi: Mt Maoershan *	SYNU 08030062	HQ228163	/
**68**	*R. luanchuanensis*	China: Henan: Luanchuan *	KIZ 047452	/	MF149923
**69**	*R. luanchuanensis*	China: Henan: Luanchuan *	KIZ 047393	/	MF149924
**70**	*R. omeimontis*	China: Sichuan: Mt Emei *	SYS a005304	MT408982	MT418644
**71**	*R. omeimontis*	China: Sichuan: Mt Emei *	SYS a005305	MT408983	MT418645
**72**	*R. sangzhiensis*	China: Hunan: Mt Tianping *	SYS a004286	MT408972	MT418634
**73**	*R. sangzhiensis*	China: Hunan: Mt Tianping *	SYS a004299	MT408974	MT418636
**74**	*R. zhengi*	China: Sichuan: Hongya: Zhangcun *	SCUM 0405190CJ	KX269206	MF149929
**75**	*R. zhengi*	China: Sichuan: Hongya: Zhangcun *	KIZ YP06057	DQ289104	/
**76**	*R. sauteri*	China: Taiwan: Kaohsiung *	SCUM 0405175CJ	KX269204	/
**77**	*R. shuchinae*	China: Sichuan: Zhaojue	CIB HUI040009	KX269210	/
**78**	*R. zhenhaiensis*	China: Zhejiang: Hangzhou	SYNU 08040100	KF020599	KF020613
**79**	*R. zhenhaiensis*	China: Zhejiang: Zhenhai *	KIZ 0803271	KX269218	JF939065
**80**	*R. zhenhaiensis*	China: Zhejiang: Fenghua	SYS a006208	MT408988	MT418650
**81**	*R. zhenhaiensis*	China: Jiangxi: Mt Tongbo	SYS a001952	MT408958	MT418620
**82**	*R. zhenhaiensis*	China: Jiangxi: Mt Guanshan	SYS a007000	MT408995	MT418657
**83**	*R. zhenhaiensis*	China: Jiangxi: Mt Yangjifeng	SYS a007422	MT409002	MT418664

### Phylogenetic analyses

For phylogenetic analyses, 26 additional sequences from all known Chinese congeners of the subgenusRana (except R. (R.) chevronta) and an out-group sequence of R. (Liuhurana) shuchinae Liu, 1950 were obtained from GenBank and incorporated into our dataset. Detailed information of these materials is shown in Table 1 and Figure 1. DNA sequences were aligned respectively by the Clustal W algorithm with default parameters (Thompson et al. 1997). For GenBank sequences that lack information for part of the segments, we filled the blank sites with “N”. The aligned data was trimmed for allowing no gap positions and default parameters in Gblocks version 0.91b (Castresana 2000). All newly obtained sequences were deposited in GenBank (Table 1). PartitionFinder2 was used to test the best partitioning scheme and jModelTest v2.1.2 was used to test the best fitting nucleotide substitution models, resulting in the best fit models for the partitions of COI and 16S as GTR + I + G. Sequenced data were analyzed using Bayesian inference (BI) in MrBayes 3.2.4 (Ronquist et al. 2012), and maximum likelihood (ML) in RaxmlGUI 1.3 (Silvestro and Michalak 2012). Two independent runs were conducted in a BI analysis, each of which was performed for 10,000,000 generations and sampled every 1000 generations with the first 25% samples discarded as burn-in, resulting in a potential scale reduction factor (PSRF) of <0.005. In ML analysis, the bootstrap consensus tree inferred from 1000 replicates was used to represent the evolutionary history of the taxa analyzed. Pairwise distances were respectively calculated b in MEGA 6 using the uncorrected *p*-distance model.

## Results

### Morphological comparison

The unnamed specimens from Jiangxi and Hunan are assigned to the *Rana japonica* group based on the following combined characteristics: digits without circummarginal grooves, and dorsolateral fold distinct, extending straight from the posterior margin of the upper eyelid to above the groin. Therefore, we compare the new species with the species of the *R. japonica* group.

The new species differs from *Rana dabieshanensis* in the following characters: head length significantly larger than head width, HW/HL 0.82 in males and 0.85 in females (vs almost equal); supratympanic fold absent (vs distinct); tympanum diameter significantly smaller than eye diameter with TD/ED = 0.63–0.87 (vs equal); relative toe lengths I < II < III < V < IV (vs I < II < V < III < IV); toe webbing formula I 1⅓ – 2 II 1⅓ – 2⅓ III 1 ½ – 2⅔ IV 3 – 1⅓ V (vs I 2 – 1 II 2^+^ – 1^+^ III 3 – 2 IV 2 – 2^+^ V); and nuptial pad creamy white in breeding males (vs gray-blackish).

The new species differs from *R. omeimontis* as follows: body size smaller, SVL = 48.2–57.5 mm in adult females (vs 61.7–70.3 mm in females); head length significantly larger than head width, HW/HL = 0.82 in males and 0.85 in females (vs head length slightly larger than head width, HW/HL = 0.94 in males and 0.92 in females); and supernumerary tubercles present below the bases of each finger (vs absent).

The new species further differs from *R. hanluica* as follows: supratympanic fold absent (vs present); toe webbing formula I 1⅓ – 2 II 1⅓ – 2⅓ III 1 ½ – 2⅔ IV 3 – 1⅓ V (vs I 1⅓ – 1⅔ II 1 – 2 III 1⅓ – 2½ IV 2⅓ – 1 V); reddish tubercles present on loreal and temporal regions in breeding males (vs absent, but white horny spines present around loreal and temporal regions, upper eyelids, and snout in breeding males). The new species differs from *R. longicrus* in having: internarial distances larger than interorbital distances (vs smaller) and toe webbing formula I 1⅓ – 2 II 1⅓ – 2⅓ III 1 ½ – 2⅔ IV 3 – 1⅓ V (vs I 1⅔ – 2⅓ II 1½ – 2⅔ III 1⅔ – 3½ IV 3⅓ – 1½ V); from *R. zhenhaiensis*: supratympanic fold absent (vs present), dorsolateral fold extending straight from posterior margin of upper eyelid to above groin (vs dorsolateral fold slightly curved above tympanum), two outer metacarpal tubercles distinctly separated (vs merged at base), tibio-tarsal articulation reaching forward beyond tip of snout (vs around nostril), and nuptial pad creamy white in breeding males (vs gray or gray-brownish); from *R. culaiensis*: dorsolateral fold extending straight from posterior margin of upper eyelid to above groin (vs dorsolateral fold slightly curved above tympanum), and tibio-tarsal articulation reaching forward beyond tip of snout (vs at nostril); from *R. jiemuxiensis*: dorsolateral fold extending straight from posterior margin of upper eyelid to above groin (vs dorsolateral fold slightly curved above tympanum), head length significantly larger than head width (vs slightly larger), internarial distances larger than interorbital distances (vs smaller), and two outer metacarpal tubercles distinctly separated (vs merged at base); from *R. chaochiaoensis*: supratympanic fold absent (vs present), internarial distances larger than interorbital distances (vs smaller), and toe webbing formula I 1⅓ – 2 II 1⅓ – 2⅓ III 1 ½ – 2⅔ IV 3 – 1⅓ V (vs I 1 – 1⅔ II 1⅓ – 2 III 1½ – 2½ IV 2⅔ – 1 V); from *R. japonica*: outer metacarpal tubercles present (vs absent), tibio-tarsal articulation reaching forward beyond tip of snout (vs reaching or beyond tip of snout in males, reaching at center of eye or beyond nostril in females), nuptial pad creamy white and divided into three parts (vs nuptial pads grayish brown or yellowish brown and divided into two parts).

From *Rana chevronta*, which lacks molecular data, the new species can be distinguished by its larger body size, SVL = 48.3–57.8 mm in adult males (vs 39.7–44.0 mm), head length significantly larger than head width (vs almost equal), relative finger lengths I < II < IV < III (vs II < IV < I < III), and nuptial pad creamy white and divided into three parts in breeding males (vs purplish gray and undivided).

### Phylogenetic analyses

**Figure 2.**
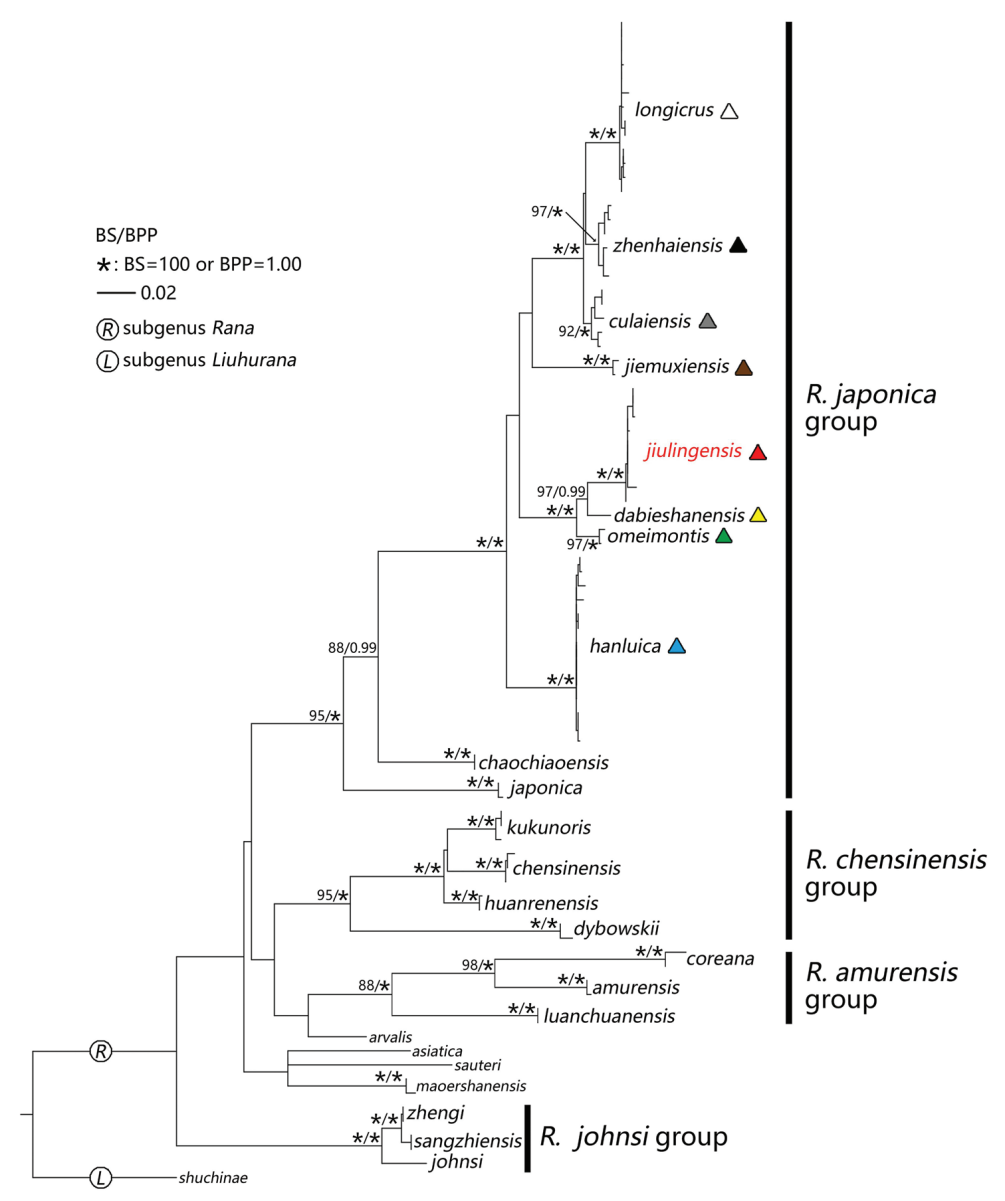
Bayesian inference and maximum-likelihood phylogenies based on mitochondrial 16S and COI genes.

The ML and BI analyses resulted in essentially identical topologies and are integrated in Figure 2, in which the major nodes are sufficiently supported with the Bayesian posterior probabilities (BPP) >0.95 and the bootstrap supports (BS) for maximum likelihood analysis >85. The pairwise distances based on COI and 16S genes among all samples are given in the Supplementary material, Tables S1 and S2, respectively.

The *Rana* samples representing the new species are grouped in a distinct and robust monophyletic lineage with high support (BPP = 1.00 and BS = 100) and low divergence (mean 0.3%, ranging 0.0–0.6% in COI, and mean 0.1%, ranging 0.0–0.5% in 16S); they form a separate evolutionary lineage within the *R. japonica* group. This lineage from Jiangxi and Hunan is close to *R. dabieshanensis* from Anhui and *R. omeimontis* from Sichuan. The smallest genetic distance between this lineage and a previously recognized species is 3.4–4.0% in COI (with *R. omeimontis*) and 1.6–2.0% in 16S (with *R. dabieshanensis*), which are significant when compared to all other recognized species (e.g. 2.8–3.6% in COI between *R. longicrus* and *R. culaiensis*; 1.2–1.3% in 16S between *R. dabieshanensis* and *R. omeimontis*).

Therefore, based on the significant morphological differences and phylogenetic divergence, these specimens from Jiangxi and Hunan represent a distinct evolutionary lineage and are described as a new species, *Rana jiulingensis* sp. nov.

### Taxonomic account

#### Rana (Rana) jiulingensis

Taxon classificationAnimaliaAnuraRanidae

Wan, Lyu & Wang sp. nov.

3C6FD03C-7B92-53A0-85DF-571C00A62689

http://zoobank.org/2E012E54-EFA3-4AA3-9B9F-0F884305AABD

##### Holotype.

**Figure 3.**
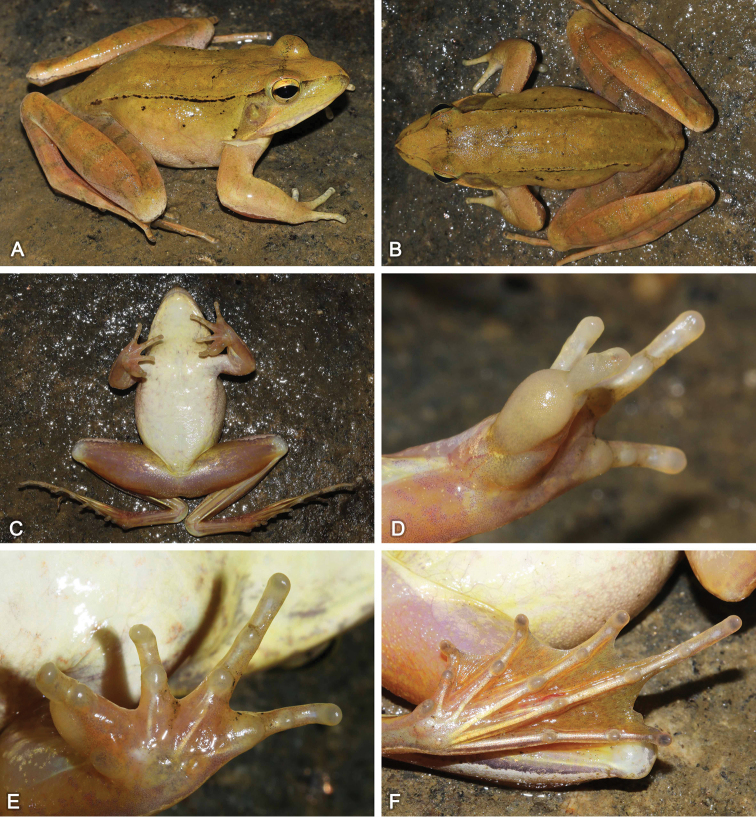
Morphological features of the adult male holotype SYS a005519 of *Rana jiulingensis* sp. nov. in life. **A** dorsolateral view **B** dorsal view **C** ventral view **D** grey nuptial pad **E** left hand **F** left foot.

SYS a005519 (Fig. 3), adult male, collected by Zhi-Tong Lyu, Jian Wang and Hai-Long He on 14 September 2016 from Guanshan Nature Reserve (28.5535N, 114.5878E; ca 300 m a.s.l.), Yifeng County, Jiangxi province, PR China.

##### Paratypes.

Seven adult specimens. Females SYS a002584–2585 collected by Jian Zhao on 8 May 2014 from Mt Wugong (27.4607N, 114.2059E; ca 1100 m a.s.l.), Anfu County, Jiangxi province. Male SYS a005511 collected by Zhi-Tong Lyu, Jian Wang and Hai-Long He on 13 September 2016 from Mt Mufu (28.9750N, 113.8304E; ca 1200 m a.s.l.), Pingjiang County, Hunan province. Males SYS a006494, SYS a006495/CIB 110014, and females SYS a006451, 6496, collected by Zhi-Tong Lyu on 5–6 August 2017 from Mt Dawei (28.4250N, 114.0805E; ca 800 m a.s.l.), Liuyang City, Hunan province.

##### Etymology.

The specific name *jiulingensis* is in reference to the type locality, Guanshan Nature Reserve in Jiuling Mountains.

##### Suggested common name.

Jiuling Mountains Brown Frog (in English), Jiu Ling Shan Lin Wa (九岭山林蛙 in Chinese)

##### Diagnosis.

*Rana jiulingensis* sp. nov. is distinguished by the following morphological characteristics: (1) body medium-sized, SVL = 48.3–57.8 (51.7 ± 4.3, *n* = 4) mm in adult males, 48.2–57.5 (50.8 ± 4.4, *n* = 4) mm in adult females; (2) head length significantly larger than head width; (3) supratympanic fold absent; (4) dorsolateral fold distinct and thin, extending straight from posterior margin of upper eyelid to above groin; (5) internarial distances larger than interorbital distances; (6) tympanum diameter significantly smaller than eye diameter, TD/ED = 0.63–0.87; (7) fingers without circummarginal grooves, unwebbed, relative finger lengths I < II < IV < III; (8) presence of supernumerary tubercles below the bases of each finger, presence of three separated metacarpal tubercles; (9) toes without circummarginal grooves, toe webbing formula: I 1⅓ – 2 II 1⅓ – 2⅓ III 1 ½ – 2⅔ IV 3 – 1⅓ V, relative toe lengths I < II < III < V < IV; (10) tibio-tarsal articulation reaching forward beyond tip of snout; (11) heels overlapping; (12) dorsal skin smooth, flanks smooth with few granules; (13) absence of vocal sacs in males; (14) breeding males possess creamy white nuptial pad with tiny hoar spines on the finger I, divided into three parts; (15) presence of reddish tubercles on loreal and temporal regions in breeding males.

##### Description of holotype.

SYS a005519, adult male, SVL 57.8 mm. Head length significantly larger than head width (HW/HL = 0.85); snout pointed and projecting; nostril closer to tip of snout than eye; canthus rostralis distinct; internasal distance slightly larger than interorbital distance; tympanum rounded, smaller than eye (TD/ED = 0.72); tympanic rim prominent; pupil horizontal; loreal region concave, sloping outwards; vomerine teeth present; tongue deeply notched posteriorly; vocal sacs absent.

Forearms 0.19 of SVL and hand 0.26 of SVL; fingers slender, without web but with narrow fringe; tip of fingers rounded, not expanded, without circummarginal grooves; relative finger lengths I < II < IV < III; subarticular tubercles significantly prominent, rounded; distinct, small, rounded supernumerary tubercles below the bases of each finger; inner metacarpal tubercle indistinct, ovoid, partly covered by nuptial pad; two outer metacarpal tubercles distinctly separated, slightly larger, long elliptic; nuptial pad with tiny spines on the finger I, divided into three parts, the basal one around the inner metacarpal tubercle and partly covering it, the largest one from the edge of the basal one to the subarticular tubercle of finger I, the smallest one extending from the edge of the biggest one to the tip of finger I.

Tibia 0.63 of SVL and foot 0.88 of SVL; heels overlapping when hindlimbs flexed at right angles to axis of body; tibio-tarsal articulation reaching forward beyond the tip of snout when hindlimb stretched along the side of the body; relative toe lengths I < II < III < V < IV; toes webbing formula: I 1⅓ – 2 II 1⅓ – 2⅓ III 1 ½ – 2⅔ IV 3 – 1⅓ V; absence of lateral fringes on the lateral edges of toes I and V; subarticular tubercles oval and distinct; inner metatarsal tubercle large, ovoid, outer metatarsal tubercle small.

Dorsal skin smooth with sparse tiny granules; several small tubercles on flank; supratympanic fold absent; dorsolateral fold distinct and thin, extending straight from posterior margin of upper eyelid to above groin; several tiny granules on the skin of loreal and temporal regions; ventral surface smooth, large flattened tubercles densely arranged on the rear of thigh and around vent.

##### Coloration of holotype.

In life, dorsal surface yellowish brown with few black spots; black speckles forming a linear stripe between eyelids; dorsolateral fold intermittently edged with black on two sides; loreal region yellowish; temporal region yellowish, slightly tinged with grey; tiny granules on loreal and temporal regions reddish; dorsal forelimbs and hindlimbs reddish with indistinct greenish grey transverse bars. Throat yellowish; chest and belly creamy white; ventral surface of forelimbs and hindlimbs flesh color; nuptial pad creamy white; tubercles around vent yellowish.

In preservative, dorsal surface turns grey with black spots and light grey patches; limbs taupe with brown transverse bars. Ventral surface white, with greyish mottling on throat and belly; ventral surface of limbs beige; hands and toe webs dark grey.

##### Variations.

**Figure 4.**
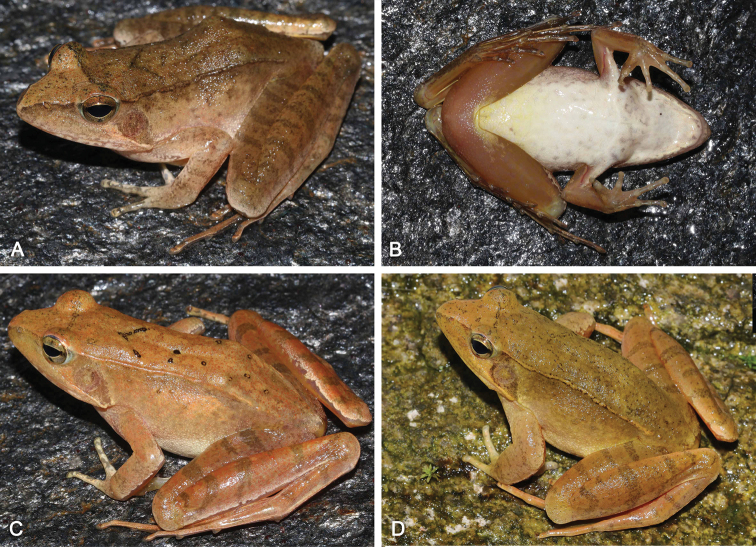
Variations of *Rana jiulingensis* sp. nov. **A, B** dorsolateral view and ventral view of male paratype SYS a006495 **C** male paratype SYS a006496 **D** male paratype SYS a00511.

Measurements of type series specimens are given in Table 2. Coloration of dorsal skin varies from brown to yellowish brown (Fig. 4). Black edges on dorsolateral fold indistinct in all paratypes. SYS a006495 and 6496 with V-shaped mark. The number of transverse bars ranges from two to five on forearms, three or four on thigh, and three to six on tibia.

**Table 2.** Measurements (in mm) of the type series of *Rana jiulingensis* sp. nov. (* = holotype).

	SYS a005519*	SYS a005511	SYS a006494	SYS a006495	SYS a002584	SYS a002585	SYS a006451	SYS a006496
Sex	Male	Male	Male	Male	Female	Female	Female	Female
SVL	57.8	51.6	48.3	49.1	57.5	48.4	49.4	48.2
HL	21.6	19.3	18.4	17.7	22.3	18.9	18.2	19.4
HW	18.4	17.0	15.7	12.6	19.3	16.1	15.5	15.8
SL	7.8	7.5	7.3	7.5	8.1	7.1	7.2	7.4
IN	4.1	3.8	3.5	3.2	4.2	3.6	4.1	4.1
IO	3.4	2.7	3.0	3.1	3.6	3.4	3.3	3.3
ED	6.3	5.5	5.2	5.0	5.2	4.4	4.7	4.6
TD	4.6	3.5	3.3	3.9	4.5	3.7	3.2	3.4
TED	1.9	2.0	1.7	2.0	1.7	1.7	1.7	1.6
HND	15.0	14.0	14.0	12.5	15.3	13.9	13.2	12.5
RAD	11.2	11.2	9.0	9.3	10.5	10.4	10.9	10.1
FTL	50.7	44.2	41.7	42.8	47.6	43.8	44.3	41.1
TIB	36.6	30.4	29.5	30.7	36.1	31.8	31.8	29.0

##### Distribution and ecology.

Currently, *Rana jiulingensis* sp. nov. is known from Guanshan Nature Reserve in the Jiuling Mountains and Mount Wugong in the Wugong Mountains of northwestern Jiangxi, and Mount Mufu and Mount Dawei in the Mufu Mountains of northeastern Hunan. This suggests that its geographic distribution is the central and northern parts of the Luoxiao Range (Fig. 1). All individuals were found on the surface of paths or on the bush leaves beside paths in subtropical evergreen broadleaved forests. Males SYS a005511 and 5519, which were collected in mid-September, bear a well-developed nuptial pad, while males SYS a006494 and 6495, collected in early August, are without a nuptial pad. This suggests that the breeding season of this species might begin in September.

## Discussion

All recognized species of the subgenusRana from China (except for *R. chevronta*) are included in our work for morphological and molecular analyses. Four monophyletic clades are supported by high values (BPP = 1.00 and BS > 85, respectively) in the phylogenetic tree. Three of them correspond to the morphologically recognized *R. japonica* group, *R. chensinensis* group, and *R. amurensis* group. The fourth, unnamed monophyletic clade includes *R. johnsi*, *R. sangzhiensis*, and *R. zhengi*. Within this unnamed clade, *R. sangzhiensis* and *R. zhengi* cluster together with significant support (BPP = 1.00 and BS = 100) and little divergence (0.0–0.4% in COI and 0.0–0.4% in 16S), which is consistent with the original morphological identification by Zheng et al. (1997). Therefore *R. zhengi* is considered a synonym of *R. sangzhiensis*. Furthermore, all species of this clade were morphologically previously assigned to *Pseudorana* (Fei et al. 2009). Thus, based on the phylogenetic relationships and morphological similarities, this monophyletic clade is proposed as a new species group, the *Rana johnsi* group. For the remaining species, their exact placements remain unresolved due to the insignificant support. Further study of these species is needed, and new species groups might be proposed for these outcast species.

Within the *Rana japonica* group, the genetic divergences among three species, *R. longicrus*, *R. zhenhaiensis*, and *R. culaiensis*, are relatively closer than other species. Additionally, the validations of these species have been supported by the morphological examinations (Li et al. 2008; Fei et al. 2009). Anuran frogs are suggested with conservative phenotypes (Cherry et al. 1978). Cryptic species, which are morphologically identical but genetically differentiated, are also common in most species complexes (e.g. Yan et al. 2011; Kuraishi et al. 2013; Xiong et al. 2015; Lyu et al. 2019, 2020). With remarkable morphological diversity, but relatively smaller genetic differentiation, *R. longicrus*, *R. zhenhaiensis*, and *R. culaiensis* show a special situation. This suggests that an integrative taxonomic approach is especially important in delimitation of anuran species, and that reliance solely on morphological or molecular evidence would be misleading.

The discovery of *Rana jiulingensis* sp. nov. increases the diversity of the genus *Rana* in the Luoxiao Range to five species (Fig. 1). This situation indicates that the Luoxiao Range has the greatest diversity of *Rana* species in southern China and may be key to speciation of the genus *Rana*.

## Supplementary Material

XML Treatment for Rana (Rana) jiulingensis

## Appendix 1

**Specimens examined**

*Rana chaochiaoensis* (3): **China: Sichuan**: Zhaojue County (type locality): SYS a001815–1816, 1831.

*Rana chensinensis* (2): **China: Henan**: Mt Yawu: SYS a002392–2393.

*Rana culaiensis* (4): **China: Jiangxi**: Mt wugong: SYS a002634; Shanggao County: SYS a002641;Mt Meiling: SYS a004239,4241.

*Rana hanluica* (35): **China: Hunan**: Mt Yangming (type locality): SYS a001137–1147; Mt Bamian: SYS a004086; Mt Badagong: SYS a004298; Mt Yunshan: SYS a004358–4359; Mt Xuefeng: SYS a007216; Suining County: SYS a007250–7251; Mt Shunhuang: SYS a007259–7260; **Guizhou**: Mt Leigong: SYS a002233; Mt Fanjing: SYS a004346; **Jiangxi**: Mt Jinggang: SYS a004195–4196; Mt Qiyun: SYS a004087; **Guangxi**: Longsheng County: SYS a002284–2288; Mt Dupangling: SYS a005086–5088; **Guangdong**: Renhua County: SYS a007009–7100.

*Rana jiemuxiensis* (2): **China: Hunan**: Jiemuxi Nature Reserve (tpye locality): SYS a004318–4319.

*Rana kukunoris* (7): **China: Sichuan**: Hongyuan County: SYS a006652–6654; Maoxian County: SYS a005381–5384.

*Rana longicrus* (18): **China: Fujian**: Mt Yashu: SYS a005892, 5905; **Guangdong**: Renhua County: SYS a000732–0735, 5624–5625; Mt Nankun: SYS a000754, 4589, 5579; Yingde City: SYS a00 7519; Pu’ning City: SYS a004605; Mt Tonggu: SYS a005808; **Jiangxi**: Mt Qiyun: SYS a002355; Mt Jiulian: SYS a004487; Mt Magu: SYS a007038; Suichuan County: SYS a007097.

*Rana omeimontis* (5): **China: Sichuan**: Mt Emei (type locality): SYS a005304–5305; Anzhou District: SYS a005393; **Guizhou**: Qixingguan District: SYS a007294–7295.

*Rana zhenhaiensis* (7): **China: Zhejiang**: Fenghua District: SYS a006208, 7506–7507; **Jiangxi**: Mt Tongbo: SYS a001951–1953; Guanshan Nature Reserve: SYS a007000.

## Supplementary materials

Supplementary material 1 Tables S1, S2 Data type Tables for genetic distances Explanation note **Table S1.** Pairwise distances based on COI gene among all sample used in this study. **Table S2.** Pairwise distances based on 16S gene among all sample used in this study. https://binary.pensoft.net/file/423007This dataset is made available under the Open Database License (http://opendatacommons.org/licenses/odbl/1.0/). The Open Database License (ODbL) is a license agreement intended to allow users to freely share, modify, and use this Dataset while maintaining this same freedom for others, provided that the original source and author(s) are credited.Han Wan, Zhi-Tong Lyu, Shuo Qi, Jian Zhao, Pi-Peng Li, Ying-Yong Wang

## References

[B1] AmphibiaWeb (2019) AmphibiaWeb. University of California, Berkeley, CA, USA. http://amphibiaweb.org [Accessed on: 2019-9-11]

[B2] Chen X Chen Z Jiang J Qiao L Lu Y Zhou K Zheng G Zhai X Liu J (2013) Molecular phylogeny and diversification of the genus *Odorrana* (Amphibia, Anura, Ranidae) inferred from two mitochondrial genes. Molecular Phylogenetics and Evolution 69: 1196–1202. 10.1016/j.ympev.2013.07.023 23911727

[B3] Cherry LM Case SM Wilson AC (1978) Frog perspective on the morphological difference between humans and chimpanzees. Science 200: 209–211. 10.1126/science.635583 635583

[B4] Fei L Ye CY Jiang JP (2012) Colored Atlas of Chinese Amphibians and their Distributions. Sichuan Publishing House of Science and Technology, Chengdu. [In Chinese]

[B5] Fei L Hu SQ Ye CY Huang YZ (2009) Fauna Sinica. Amphibia Vol. 2 Anura. Science Press, Beijing. [In Chinese]

[B6] Frost DR (2020) Amphibian Species of the World: an Online Reference. Version 6.0. American Museum of Natural History, New York, USA. http://research.amnh.org/herpetology/amphibia/index.html [Accessed on: 2020-4-29]

[B7] Hu SQ Fei L Ye CY (1978) Three new amphibian species in China. Materials for Herpetological Research 4: 20.

[B8] Kuraishi N Matsui M Hamidy A Belabut DM Ahmad N Panha S Sudin A Yong HS Jiang JP Ota H Thong HT Nishikawa K (2013) Phylogenetic and taxonomic relationships of the *Polypedates leucomystax* complex (Amphibia). Zoologica Scripta 42: 54–70. 10.1111/j.1463-6409.2012.00562.x

[B9] Li PP Lu YY Li A (2008) A new species of brown frog from Bohai, China. Asiatic Herpetological Research 11: 60–68.

[B10] Liu CC (1946) A new woodfrog *Rana chaochiaoensis* with a discussion of its allied species, from West China. Journal of the West China Border Research Society, Series B 16: 7–14.

[B11] Liu MY Zhang SQ Liu M (1993) A new species of Ranidae from Liaoning, China (Anura). Acta Zootaxonomica Sinica 18: 493–497.

[B12] Lu YY Li PP Jiang DB (2007) A new species of *Rana* (Anura, Ranidae) from China. Acta Zoologica Sinica 32: 792–801.

[B13] Lyu ZT Huang LS Wang J Li YQ Chen HH Qi S Wang YY (2019) Description of two cryptic species of the *Amolops ricketti* group (Anura, Ranidae) from southeastern China. ZooKeys 812: 133–156. 10.3897/zookeys.812.29956 PMC6328530 30636913

[B14] Lyu ZT Dai KY Li Y Wan H Liu ZY Qi S Lin SM Wang J Li YL Zeng YJ Li PP Pang H Wang YY (2020) Comprehensive approaches reveal three cryptic species of genus *Nidirana* (Anura, Ranidae) from China. ZooKeys 914: 127–159. 10.3897/zookeys.914.36604 32132857 PMC7046709

[B15] Meyer CP Geller JB Paulay G (2005) Fine scale endemism on coral reefs: archipelagic differentiation in turbinid gastropods. Evolution 59: 113–125. 10.1111/j.0014-3820.2005.tb00899.x 15792232

[B16] Ronquist F Teslenko M Van Der Mark P Ayres DL Darling A Höhna S Larget B Liu L Suchard MA Huelsenbeck JP (2012) MrBayes 3.2: efficient Bayesian phylogenetic inference and model choice across a large model space. Systematic Biology 61: 539–542. 10.1093/sysbio/sys029 22357727 PMC3329765

[B17] Savage JM (1975) Systematics and distribution of the Mexican and Central American stream frogs related to *Eleutherodactylus rugulosus*. Copeia 2: 254–306. 10.2307/1442883

[B18] Shen Y Jiang J Yang D (2007) A new species of the genus *Rana*–*Rana hanluica* sp. nov. from Hunan Province, China (Anura: Ranidae). Acta Zoologica Sinica 53: 481–488.

[B19] Simon C Frati F Beckenbach A Crespi B Liu H Flook P (1994) Evolution, weighting, and phylogenetic utility of mitochondrial gene sequences and a compilation of conserved polymerase chain reaction primers. Annals of the Entomological Society of America 87: 651–701. 10.1093/aesa/87.6.651

[B20] Silvestro D Michalak I (2012) RaxmlGUI: a graphical front-end for RAxML. Organisms Diversity and Evolution 12: 335–337. 10.1007/s13127-011-0056-0

[B21] Stejneger L (1898) On a collection of batrachians and reptiles from Formosa & adjacent islands. Journal of the College of Science 12: 215–225.

[B22] Tamura K Stecher G Peterson D Filipski A Kumar S (2013) MEGA6: molecular evolutionary genetics analysis, version 6.0. Molecular Biology and Evolution 30: 2725–2729. 10.1093/molbev/mst197 24132122 PMC3840312

[B23] Thompson JD Gibson TJ Plewniak F Jeanmougin F Higgins DG (1997) The CLUSTAL_X windows interface: flexible strategies for multiple sequence alignment aided by quality analysis tools. Nucleic Acids Research 25: 4876–4882. 10.1093/nar/25.24.4876 9396791 PMC147148

[B24] Wang C Qian L Zhang C Guo W Pan T Wu J Wang H Zhang B (2017) A new species of *Rana* from the Dabie Mountains in eastern China (Anura, Ranidae). ZooKeys 724: 135–153. 10.3897/zookeys.724.19383 PMC5769725 29362536

[B25] Wang JQ Li PP Lu YY Dong BJ Zhou ZY Yu FL (2006) Description and comparison of tadpoles of *Rana huanrenensis* and *R. dybowskii*. Sichuan Journal of Zoology 45: 349–353. [In Chinese]

[B26] Xiong R Li C Jiang J (2015) Lineage divergence in *Odorrana graminea* complex (Anura: Ranidae: Odorrana). Zootaxa 3963: 201–229. 10.11646/zootaxa.3963.2.3 26249398

[B27] Yan F Jiang K Chen H Fang P Jin J Li Y Wang S Murphy RW Che J Zhang Y (2011) Matrilineal history of the *Rana longicrus* species group (*Rana*, Ranidae, Anura) and the description of a new species from Hunan, southern China. Asian Herpetological Research 2: 61–71. 10.3724/SP.J.1245.2011.00061

[B28] Yang BT Zhou Y Min MS Matsui M Dong BJ Li PP Fong JJ (2017) Diversity and phylogeography of Northeast Asian brown frogs allied to *Rana dybowskii* (Anura, Ranidae). Molecular Phylogenetics and Evolution 112: 148–157. 10.1016/j.ympev.2017.04.026 28476494

[B29] Ye CY Fei L Hu SQ (1993) Rare and economic amphibians of China. Sichuan Publishing House of Science and Technology, Chengdu. [In Chinese]

[B30] Ye CY Fei L Matsui M (1995) Taxonomic studies of Chinese *Rana japonica* Guenther. Acta Herpetologica Sinica 4: 82–87.

[B31] Yuan ZY Zhou WW Chen X Poyarkov Jr. NA Chen H-M Jang-Liaw N-H Chou WH Matzke NJ Iizuka K Min M-S Kuzmin SL Zhang YP Cannatella DC Hillis DM Che J (2016) Spatiotemporal diversification of the true frogs (genus *Rana*): a historical framework for a widely studied group of model organisms. Systematic Biology 65: 824–842. 10.1093/sysbio/syw055 27288482

[B32] Zhao H Yang J Wang C Li P Yuan Z (2017) A new species of the genus *Rana* from Henan, central China (Anura,Ranidae). ZooKeys 694: 95–108. 10.3897/zookeys.694.12513 PMC5672780 29134001

[B33] Zheng MQ Fei L Jiang JP Xie F (1997) A preliminary research on the relations between ecological environment and the status of herpetological resources in Wawushan Mountain National Forest Park. Cultum Herpetologica Sinica 6–7: 67–74.

[B34] Zhou Y Wang SR Zhu HD Li PP Yang BT Ma JZ (2017) Phylogeny and biogeography of south Chinese brown frogs (Ranidae, Anura). PloS ONE 12: e0175113 10.1371/journal.pone.0175113 PMC5378408 28369142

[B35] Zhou Y Yang BT Li PP Min MS Fong JJ Dong BJ Zhou ZY Lu YY (2015) Molecular and morphological evidence for *Rana kunyuensis* as a junior synonym of *Rana coreana* (Anura: Ranidae). Journal of Herpetology 49: 302–307. 10.1670/13-111

